# Case report: Peritonitis secondary to traumatic bowel perforation during second-trimester surgical abortion

**DOI:** 10.1016/j.ijscr.2024.110065

**Published:** 2024-07-20

**Authors:** Nesrine Souayeh, Hadhami Rouis, Amal Chermiti, Amira Lika, Chaouki Mbarki, Hajer Bettaieb

**Affiliations:** Department of Gynecology and Obstetrics, Hospital of Ben Arous, Tunisia; Faculty of Medicine of Tunis, University of Tunis El Manar, Tunisia

**Keywords:** Case report, Induced abortion, Second trimester, Uterine perforation, Bowel injury

## Abstract

**Introduction and importance:**

Uterine perforation and bowel injury are rare but potentially life-threatening complications of surgical abortion. Early diagnosis results in easier management and better prognosis. We report here a case of a 39-year-old presented with peritonitis secondary to traumatic bowel perforation after second-trimester surgical abortion.

**Case presentation:**

A 39-year-old Gravida 3 Para 2 presented with acute abdominal pain two days after second trimester induced abortion. On physical examination, the patient was febrile and hypotensive with diffuse abdominal tenderness. Emergency abdomino-pelvic-CT showed generalized peritonitis with pneumoperitoneum. The patient underwent an emergency laparotomy. Per operative exploration revealed a perforation of the fundus of the uterus and the sigmoid portion of the large intestine, resulting in stercoral peritonitis. We proceeded with thorough cleansing of the abdominal cavity with physiological serum, followed by partial colectomy including the perforated sigmoid and a Hartmann's procedure. The patient was admitted to the post-operative intensive care unit for 18 days and discharged on day 27 after the surgery. Intestinal continuity restoration was performed six months after the surgery.

**Clinical discussion:**

Given the severity of second trimester pregnancy termination complications, efforts should be made to promote contraception and medical first-trimester pregnancy termination. Any unusual symptom after surgical induced abortion should lead to suspect uterine perforation.

**Conclusion:**

Uterine perforation during induced abortion is usually asymptomatic and can generally be managed conservatively. However, bowel injury may result in peritonitis, requiring immediate laparotomy and resection of perforated bowel. CT-scans can help diagnose this rare complication.

## Highlights

• Uterine perforation secondary to induced abortion is a rare complication
• Computed tomography is the recommended whenever traumatic injury to the surrounding organs is suspected following surgical induced abortion.
• Surgical management of uterine perforation is necessary when injury to other organs is suspected.

## 1 Introduction

Roughly 121 million unintended pregnancies occurred each year between 2015 and 2019. Of these pregnancies, 61 % ended in induced abortion, translating in 73 million abortions per year, most off which occur in low and middle income countries [1]. In developed countries, legal termination of pregnancy (TOP) is relatively safe [2], with a mortality rate of 10 per 100,000 procedures [1,3]. Although the overall risk of mortality related to induced abortion is low, this risk increases exponentially by 38 % for each additional week of gestation [4]. In Tunisia, voluntary pregnancy termination is legal in the first trimester, and can be authorized later in the pregnancy when dictated by a medical condition [5]. In this case, we report a rare complication of surgical second trimester induced abortion. This work has been reported in line with the SCARE criteria [6].

## 2 Case presentation

**Fig. 1:**
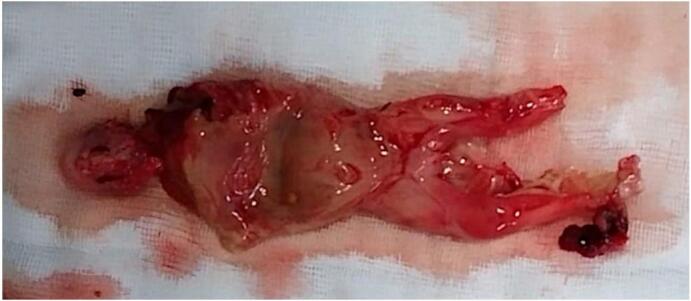
Remains of the foetus found in the abdominal cavity during laparotomy.

**Fig. 2:**
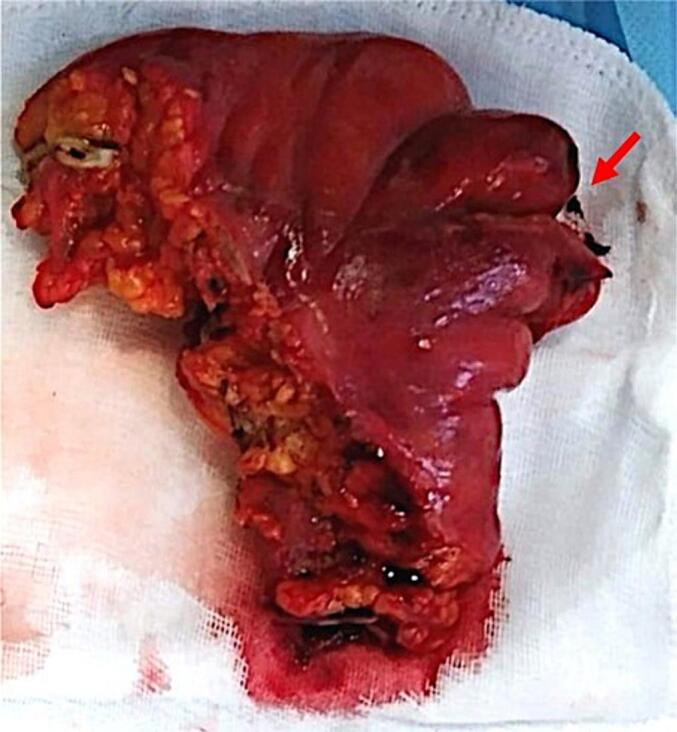
Surgical specimen of the colectomy including the sigmoid. The red arrow indicates the perforation site.

A 39-year-old woman, Gravida 3 Para 2, presented to our emergency room with acute abdominal pain. The patient had no medical history except for two caesarean deliveries. She underwent an illegal second-trimester voluntary pregnancy termination, by cervical dilation and instrumental extraction two days earlier. A licenced gynaecologist performed the procedure in a private clinic. The patient complained about fever and diarrhoea during the last 24 h. On physical examination, the patient was febrile and hypotensive with diffuse abdominal tenderness. Blood work showed an elevated white cell count of 21,000/mm^3^ along with a high C-reactive protein (CRP) level of up to 279 mg/L. An emergency abdomino-pelvic computed tomography scan was performed, revealing generalized peritonitis with pneumoperitoneum. After prompt resuscitation, the patient underwent an emergency laparotomy performed by a senior general surgeon and gynaecologist. Per operative exploration revealed a perforation of the fundus of the uterus and the sigmoid portion of the large intestine, resulting in stercoral peritonitis. We also found the remains of the foetus in the abdominal cavity (Fig. 1). We proceeded with thorough cleansing of the abdominal cavity with physiological serum, followed by partial colectomy including the perforated sigmoid (Fig. 2) and a Hartmann's procedure. The uterine perforation was not sutured as there was no bleeding, and the wound margins were sunken. The immediate post-operative follow-up was complicated by a Douglas-fir abscess, successfully managed by trans-vaginal evacuation and broad-spectrum antibiotics. The patient was admitted to the post-operative intensive care unit for 18 days and discharged on day 27 after the surgery. Intestinal continuity restoration was performed six months after the surgery.

## 3 Patient's perspective

The patient expressed that this experience has changed her life. The physical pain has been excruciating and the mental toll has been enormous. The patient was concerned about the long-term effects on her health and her ability to care for her children. The support of her family, friends, and healthcare providers has been essential in getting her through this difficult time.

## 4 Discussion

Second trimester termination of pregnancy best method is highly controversial, mainly regarding adverse outcomes and complications [3]. However, only few studies compared surgical and medical second trimester abortion and randomized comparison has proven difficult to carry out [3,7,8]. In developed countries, second trimester induced abortion is performed essentially medically using the association of mifepristone and misoprostol [7]. Although medical abortion during second trimester has been shown to be effective and acceptable [7,9], it is associated with higher risk of complications when compared with dilatation and evacuation [7,8]. However, surgical abortion after 15 weeks of gestation depends on the surgeon performing the procedure skills [7,8].

Uterine perforation is the most common site of upper genital tract injury during surgical induced abortion [10]. It may be accompanied by surrounding organs injuries, including the bowel, bladder, and surrounding vasculature [10,11]. Data on these injuries is scarce, with three case series of 92 total uterine perforations reporting bowel or bladder injury in six cases [12]. Overall, uterine perforation is uncommon, with rates ranging between 0.1 and 2.3 % in safe medical abortions [[12], [13], [14]]. The risk factors of uterine perforation are mainly unsafe abortion and inexperience of the surgeon [15]. Other factors may affect the accessibility to the uterine cavity such as cervical stenosis and uterine anteflexion/retroflexion or fragilize the myometrium like uterine scaring, all of which increase the risk of uterine perforation especially in the second trimester [[16], [17], [18]]. Patients with uterine perforation typically present with severe and continued pelvic pain in contrast with the expected mild-to-moderate pelvic cramping after uncomplicated procedure [12,19]. However, many patients can present with a delayed clinical symptoms, weeks after the procedure, depending on the location of the uterine perforation and the association with other organs injuries [20,21].

Focal or diffuse abdominal/pelvic pain, abdominal distension, heavy or persistent vaginal bleeding, haematuria, and fever are all symptoms that should lead clinicians to suspect uterine perforation [12]. The presence of tachycardia and hypotension on physical examination should first and foremost evoke septic or haemorrhagic shock [22].

Ultrasound examination could be useful to confirm the diagnosis of uterine perforation, showing the defect in the myometrial wall with or without abdominal free fluid [12,23]. However, if the ultrasound is inconclusive, it should not rule out uterine perforation [12]. Abdomino-pelvic computed tomography is recommended whenever bowel perforation is suspected [[24], [25], [26]]. It has a high sensitivity and specificity to detect and localize the site of perforation [[24], [25], [26]]. Gastro-intestinal perforation often manifests with extraluminal gas, wall discontinuity or thickening, and fat stranding [24].

Uterine perforation with bowel injury requires surgical management proceeded by proper resuscitation and symptomatic management, including intravenous fluid perfusion and broad-spectrum antibiotics [12]. The surgery includes often the resection of damaged bowel while anastomosis is not always possible, especially in the presence of peritonitis. In the case of delayed diagnosis, the management is even more challenging, and the prognosis is worse [27]. Despite the serious nature of these injuries, patients who receive prompt surgical intervention typically have favourable outcomes [28].

## 5 Conclusion

Uterine perforation during induced pregnancy termination, especially in the second trimester, can lead to severe complications such as bowel injury and peritonitis. Although CT scans can help diagnose this rare complication, medical professionals should always have a high clinical suspicion of uterine perforation when treating patients who have had a previous abortion. Careful preoperative assessment, use of ultrasonography, and skilled surgical technique are critical in minimizing the risk of these adverse events.

## Consent

Written informed consent was obtained from the patient for publication and any accompanying images. A copy of the written consent is available for review by the Editor-in-Chief of this journal on request.

## Ethical approval

This case report was approved by the local ethics committee of our institution under the number 03/2024. Written informed consent was obtained from the patientfor publication and any accompanying images. A copy of the written consent is available for review by the Editor-in-Chief of this journal on request.

## Funding

Not applicable.

## Author contribution

Drafting the article: Nesrine Souayeh, Hajer Bettaieb, Amal Chermiti

Acquisition of data: Hadhami Rouis, Amira lika, Amal Chermiti

Revising the article: Nesrine Souayeh, Hajer Bettaieb, Chaouki Mbarki

All the authors have read and agreed to the final manuscript.

## Guarantor

Nesrine Souayeh

## Research registration number

Not applicable.

## Conflict of interest statement

The authors declared that they have not received any funding.
